# Indigenous Peoples’ human genomic sovereignty: Lessons for Africa

**DOI:** 10.1111/dewb.12466

**Published:** 2024-10-15

**Authors:** Faith Kabata

**Keywords:** Canada, human genomic research, indigenous peoples, indigenous peoples’ human genomic sovereignty, right to science, USA

## Abstract

Human genomics research with indigenous peoples has often been characterised by tension between the ‘western’ science ideologies and indigenous peoples’ cultural beliefs in relation to their human genetic resources and data. This article explores this tension from the lens of the concept of indigenous peoples’ human genomic sovereignty and tests the applicability of the concept in Africa. The article achieves this by first highlighting the tension between ‘western’ science and indigenous peoples through three case studies from Canada, the USA, and South Africa. It then analyses indigenous peoples’ human genomic sovereignty in the USA and Canada and compares it with the notion of indigenous peoples’ sovereignty in Africa. The article concludes by highlighting lessons that indigenous groups in Africa can draw from the USA and Canada in their quest for human genomic sovereignty.

## 1 INTRODUCTION

Human genomic research in relation to indigenous peoples is often described using two competing narratives: research inequities and research exploitation. Research inequities implies the lack of diversity in human genomic research as indigenous peoples are often underrepresented, hence available genomic data predominantly represents non‐indigenous populations. Conversely, research exploitation refers to claims of top‐down and outside‐in approaches to human genomic research with indigenous peoples and the use of their human genetic resources for non‐consensual secondary research. Central to these competing narratives are indigenous peoples’ claims to regulate research and generation of knowledge about their populations and resources by invoking sovereignty over their human genetic resources. The Human Genome Diversity Project (HGDP) best exemplifies this scenario. HGDP's overarching goal was to improve diversity and inclusion in research on the human species by studying the genomes of the ‘genetic isolates’, that is, indigenous genomes. It proposed to study genetic material from 400 to 500 isolated and culturally distinct groups, including indigenous peoples.^1^ This invoked the exploitation narrative setting the HGDP in collision with indigenous peoples and bringing to the fore existing tensions between ‘western’ science ideology and the ideologies of indigenous peoples on control, use and ownership of human genetic resources.^2^ Illustratively, indigenous groups throughout the world filed no less than 30 declarations denouncing the HGDP.^3^

These tensions between ‘western’ science ideology and the beliefs and ideologies of indigenous peoples predated the HGDP and have their antecedents in prior human genomic research studies on indigenous people. Western science has so far presented human genomic research, particularly the Human Genome Project, as universal and as a project that unites all humanity.^4^ Therefore, knowledge generated about the human species at the collective level is a common resource for all and to benefit humanity as a whole.^5^ However, indigenous peoples consider their indigenous genome to be sacred and part of their ‘cultural and genetic heritage’ thus staking sovereignty claims.^6^ Further, for indigenous peoples, acquisition and utilization of human genomic knowledge may pose potential harms to them, for instance through distortion of their cultural identity and its construction.^7^ Three case studies on human genomic research involving indigenous peoples illustrate these tensions: the Havasupai tribe of Arizona in the USA; the Nuu‐chaah‐nulth tribe of British Columbia, Canada; and the San people of Southern Africa.

Two caveats are necessary: regarding the San people case; and Africa as the unit of analysis. First, on the San people case, although we consider it in this article alongside the other two case studies, literature is inconclusive with some authors depicting it as one in which indigenous peoples’ concerns were properly taken into account in the human genomic research process. The case study is however instructive in putting in perspective the contextual realities of indigenous peoples in Africa, which is central to this article. Second, on the justification for using Africa as the unit of analysis, the article acknowledges the differences relating to indigenous peoples in sub‐Saharan Africa and those in North Africa, including contrasts on the nature of claims asserted against the state.^8^ Even then, the article argues that the regional human rights standards on indigenous peoples in Africa, which the article relies on and which cover the scope a broad spectrum of rights which accrue to them such as land rights, political recognition and cultural integrity, apply to all self‐identified indigenous peoples on the continent regardless of geographical spread.

In this article, we examine this tension through the lens of indigenous peoples’ human genomic sovereignty by exploring how divergent human genomic research case studies have shaped the contours of indigenous peoples’ human genomic sovereignty and test its application in Africa where the exercise of indigenous peoples’ sovereignty is greatly inhibited by lack of political recognition. The article is organised as follows: (i) first, drawing from the Havasupai, the Nuu‐chaah‐nulth and San people case studies we illustrate the tensions between western science and indigenous peoples in human genomic research; (ii) second, we situate indigenous peoples’ genomic sovereignty claims within the broader international law discourse on indigenous peoples’ sovereignty and highlight its practical application in the form of tribal and aboriginal sovereignty in the USA and Canada and compare it with the notion of indigenous peoples’ sovereignty in Africa; (iii) third and final we Africa and extrapolate lessons for the exercise of indigenous peoples human genomic sovereignty in Africa. The article applies a qualitative approach in which it conducts an in‐depth content analysis of indigenous peoples’ sovereignty in human genomics research and draws lessons for its application in the African context.

## 2 HUMAN GENOMIC RESEARCH INVOLVING INDIGENOUS PEOPLES: CONTESTATIONS

### 2.1 Havasupai tribe of Arizona

In the Havasupai case, the main contention was the use of blood samples taken from the tribe members for secondary studies not authorized by the tribal council and granting of unauthorized access of the samples and data to other researchers.^9^ In May 1990 the Havasupai tribal council authorized researchers from Arizona State University (ASU) to conduct research on whether there was a genetic link between Havasupai genes and diabetes, which was widespread among the tribe members. Consequently, in June 1990 the researchers collected blood samples from approximately 100 Havasupai tribe members. The consent forms indicated that the blood samples were to ‘study the causes of behavioral/medical disorders’.^10^ However, no written informed consent was sought from the members and the researchers only alluded to diabetes research, hence for the tribe members their blood samples were restricted to the study of diabetes.^11^ The research failed to establish a genetic link to diabetes and was abandoned in 1991 without any communication to the Havasupai tribe on the conclusions.

Further, blood samples were collected between 1991 and 1994 from about 130 members of the Havasupai tribe with which the researchers at ASU conducted more studies on schizophrenia and inbreeding.^12^ In addition, access to DNA obtained from the cell‐lines of the original samples was provided to researchers from other universities to study ancient human migration and kidney disease.^13^ Moreover, multiple researchers were granted access to the Havasupai genetic data and published a number of articles.^14^ In 2003, the Havasupai learnt that their blood samples were used for research studies outside diabetes without their approval.^15^ According to the Havasupai tribe, the secondary use of their blood without approval for research in schizophrenia, inbreeding and human migration violated the tribe's informed consent and the research findings on schizophrenia and migration violated their cultural and religious heritage by disrupting the cultural construction of their own histories, while those on inbreeding were demeaning to the tribe.^16^

Consequently, the Havasupai issued an order banning ASU employees from their tribal land. A 2003 joint and independent investigation agreed upon between ASU and the Havasupai tribe established that numerous research studies across the USA had been conducted using the Havasupai blood samples resulting in 23 research publications out of which only 8 related to research on diabetes, while the other 15 related to research on schizophrenia, inbreeding and human migration.^17^ The Havasupai tribe lodged unsuccessful claim notices with ASU, following which in 2004, two lawsuits were filed against the Arizona Board of Regents (ABOR) and the principal researchers at ASU. The first lawsuit was filed by 52 research participants whose blood samples had been obtained, while the second was filed by the tribe as the protector of the Havasupai people. The main claims in the lawsuits were breach of fiduciary duty, lack of informed consent, fraud and misrepresentation and conversion, for which the Havasupai tribe sought $60 million compensation.^18^ In 2010, the two law suits were settled out of court with the ABOR paying the plaintiffs $700,000, returning all the remaining blood samples and research documents derived from the blood samples and a five year joint project between ASU and the Havasupai on education, economic development and health services.^19^

### 2.2 Nuu‐chaah‐nulth tribe of British Columbia

“We were of the understanding that we would have the results of the study within a year, but he never told us anything. He disappeared. He published more than 200 papers…because he was carrying our blood with him. He used us as cheap guinea pigs and that incenses me.”^20^

In the Nuu‐chaah‐nulth case, the contention was also unauthorised use of the Nuu‐chaah‐nulth samples for secondary research not approved by the tribe and the unauthorised access of their blood samples by other researchers and research institutions.^21^ In 1983, a researcher from the University of British Columbia approached the tribal leaders of the Nuu‐chaah nulth proposing a study to establish whether there was any genetic link to rheumatoid arthritis, of which two thirds of the tribe members had been diagnosed.^22^ In 1985, the researcher collected 833 vials of blood from the Nuu‐chaah‐nulth tribe members.^23^ The leaders of the tribe and each individual involved signed written consent forms for the study on arthritis, and the collection of blood samples.^24^ The researcher failed to establish a genetic link to the rheumatoid arthritis, and in 1986 he moved to University of Utah and carried with him the tribe's blood samples, which were subjected to further study to try and establish a genetic link to arthritis.^25^ In 1996, when the researcher moved to the University of Oxford, he again carried the blood samples with him which were availed to other researchers for studies on viruses caused by intravenous drug use and on population genetics.^26^

In 2000 following publication of an article on genetic ancestry, the Nuu‐chaah‐nulth tribe learnt that their blood samples had been used for research outside arthritis, and by researchers not approved by the tribal leadership.^27^ The Nuu‐chaah‐nulth protested to the University of British Columbia resulting in the return of the Nuu‐chaah‐nulth documents and blood samples to the University of British Columbia in January 2004.^28^ Subsequently, the Nuu‐chaah‐nulth tribe established the Nuu‐chaah‐nulth Research Ethics Committee to control research on their members, and in their territories.^29^ At the national level, the Nuu‐chaah‐nulth case led to the development of guidelines on research with indigenous people by the Canadian Institutes of Health Research.^30^

### 2.3 The San people of Southern Africa

In line with the caveat alluded to earlier, literature on the San people case is inconclusive. However, the case study is significant in highlighting the unique contextual realities of indigenous peoples in Africa as lacking political recognition, dispersed across nation‐states, hence weak or non‐existent collective coordination resulting to lack of clarity on who represents the indigenous authority within different groups. McInnes writing on the importance of culture in research with indigenous populations contrasted the San people case study with both the Havasupai and Nuu‐chaah‐nulth case studies noting that the San people study adhered to the ethical requirements of informed consent in research with and on indigenous peoples and that their categorisation as hunter‐gatherer was adequately addressed by the researchers.^31^ Botes, in subsequent independent research on informed consent on the genome research involving the San community of South Africa, however noted that the four San indigenous research participants could not be expected to understand the complexities of genomic research. The author thus argued that the indigenous research participants ought to have been afforded specific protection by the researchers obtaining added consent from the San Council.^32^

The perceived exploitation in the research study was raised by the San leadership. The San leadership ambiguously refers to a collection of three organisations: the Working Group on Minorities in Southern Africa (WIMSA); the South African San Council; and the South African San Institute.^33^ WIMSA, a Namibia based regional non‐governmental organisation established in 1996, was until 2016 working on the rights of the San people of Namibia, South Africa, Angola, Botswana, Zambia and Zimbabwe,^34^ while the South African San Council is a not for profit organisation whose scope is geographically limited to protection and promotion of the rights of the San people of South Africa.^35^ This unclear nature of the San leadership foregrounds the issue of lack of collective coordination of the San people and the legitimacy of the San leadership as the custodian of the collective rights/interests and representative of the indigenous authority.

According to the San leadership, the perceived controversy related to the integrity of the informed consent obtained from the San research participants who represented indigenous peoples of the Kalahari Desert and the characterization and publication of the results findings in a manner that demeaned and breached the privacy of the San people.^36^ In 2009, researchers from the Universities of Penn State, Limpopo and New South Wales sequenced the human genome of one indigenous San individual from Namibia and one individual of Bantu origin from South Africa as well as obtained the exome sequences of three other indigenous San individuals.^37^ The aim of the research was to generate a full human sequence genome of indigenous hunter‐gatherer peoples of Southern Africa, who are the oldest ancestors of modern man, and to compare the indigenous hunter‐gatherer genome with that of a Southern Africa Bantu.^38^ According to the researchers, the research was approved by the ethical review boards of the three participating universities as well as the provincial government in South Africa and also permission was granted by the Ministry of Health and Social Services in Namibia for collection of human DNA of the indigenous hunter‐gatherer peoples.^39^ The research participants consisted the four eldest members of the indigenous hunter‐gatherer communities in Namibia and one prominent South African national who represented the two largest African Bantu groups. Of the five research participants, only the South African of Bantu extraction gave written consent, while the four indigenous San peoples, who were each above 80 years, and the eldest members of their community consented through a verbal video recording.^40^ Expectedly, for the four San people, as the eldest members of their communities’ written consent was not possible owing to their inability to read and write. Botes further noted that the main obstacle was language barrier as the San research participants could not understand English, which was the researchers’ language of consent process, thus it is unlikely that they understood what they were consenting to.^41^

In February 2010, following publication of the research results and their circulation across the globe and in Southern Africa,^42^ WIMSA engaged the authors requesting to be furnished with details on the consent process, and protesting that their approval was not sought for the study.^43^ After receiving no response from the authors, the WIMSA contacted *Nature* objecting to the publication on the basis of the alleged failure of the researchers to comply with ethical guidelines.^44^

The San leadership deemed the research exploitative based on use of the terms ‘bushmen’, ‘hunter‐gatherer’ and ‘Khoisan’ which were demeaning to the San people as they portrayed them as being of low and inferior status. This concern was subsequently addressed by the authors who explained that the Namibian research participants picked the terminology as their group identifier and did not view it as demeaning.^45^ On informed consent, the San leadership claimed that the researchers should have obtained collective consent from them, over and beyond the individual consent obtained from the research participants.^46^ This claim once again raises the issue of the legitimacy of the San leadership as the representative of the indigenous authority of the San people, and in particular recognition of this alleged mandate of the San leadership by the San indigenous peoples. On this issue of recognition of the mandate of the San leadership by the San people, Botes in her explorative research found that members of the San community of South Africa exercise individual autonomy in decision‐making without any reference to or approval from the San Council.^47^ Finally, in relation to publication of the research findings, the San leadership claimed that the published results extended beyond the human genome sequence findings to conclusions which were demeaning to the San people and breached their privacy.^48^ In response, and to address the alleged exploitation in future human genomics research, the San leadership engaged with genetics, lawyers and ethicists to develop the San Code of Research Ethics, 2017.^49^ This Code requires that researchers who conduct research with the San people adhere to the values of respect, fairness, care and honesty and seek community approval.^50^ However, it is unclear how the Code is to be implemented in view of the fact that the San Council scope of operation is limited to South Africa, while San people are scattered across Southern Africa, once again posing the question: who is the representative of the indigenous authority of the San people of Southern Africa?

The foregoing highlights the unique aspects of indigenous peoples in Africa alluded to earlier in this section. As shown in the San people case study, indigenous peoples in Africa lack fixed and exclusive territory and are mainly settled as scattered population groups across various countries in Southern Africa. This results in lack of collective coordination, thus weak self‐governance mechanisms that are not recognised by and within the nation‐state, and even among the indigenous peoples.

### 2.4 Contestations arising from the case studies

The contestations raised in the three cases above can be summarised in the following broad themes: (i) initiation of research studies; (ii) management of the research process; (iii) publication of research findings; and (iv) termination of the research studies. While these concerns would arise in the context of human genomic research on non‐indigenous peoples, for indigenous peoples the cultural and spiritual beliefs associated with their human genetic resources requires certain cultural and contextual sensitivity. On initiation of the research studies, the alleged contention as alluded to by the San leadership is disregard of indigenous culture as collective permission to initiate research studies ought to have been obtained from them. In relation to management of the research process the contestations point to integrity of informed consent and consent for secondary use of samples; and the ability of indigenous peoples’ leadership structures to govern the research process. The Havasupai and Nuu‐chaah‐nulth cases raised the issue of consent for secondary use of blood samples for studies that the leadership of the tribes did not consent to. In the context of indigenous people, issues of cultural sensitivity with regard to certain research studies have arisen as certain studies are deemed culturally inappropriate, and an extension of the colonial enterprise as their findings threaten to rewrite and reconstruct their histories. In addition, in the Havasupai and the San people cases the integrity of the informed consent arose, stemming from lack of proper articulation between the researchers and the tribe members on what studies the tribe members were consenting to in the Havasupai case and in the San people the contestation that the indigenous research participants were not in a position to grant informed consent as they were illiterate and could not understand the nature of the scientific study they were to participate in. Contestation on the publication of research findings stems from interpretation of the research of findings without cultural and contextual background, hence depicting indigenous peoples in demeaning ways that further marginalise and oppress them. Accordingly, in the San people case, the failure of the researchers to consult the San leadership before publication of their research findings resulted in further marginalisation of the San people as they were portrayed as of low status. In the Havasupai and Nuu‐chaah‐nulth cases, publication of the research findings from the unauthorised secondary studies also demeaned the tribe members as the studies were culturally inappropriate. Finally, on termination of research studies, both the Havasupai and Nuu‐chaah‐nulth cases did not receive any communication on the outcome of the studies that they had consented to, which also resonates with the narrative of exploitation as the groups did not benefit from research studies on them. Further, based on indigenous peoples’ sovereignty claims over their human genetic resources, both the Havasupai and Nuu‐chaah‐nulth cases required the researchers to return the samples to the tribe.

The table below presents a summary of the contestations. Table 1

**Table 1:** Tabulated summary of the contestations of indigenous peoples’ in human genomic research contestations.

	Contestations	Havasupai tribe	Nuu‐chaah‐nulth tribe	San people
1.	Initiation of research studies	‐ (the Havasupai tribe leaders initiated the research study with the Arizona University researchers)	‐ (the Nuu‐chaah‐nulth tribal leaders initiated the research study with the researcher from University of British Columbia)	The San peoples leadership was not consulted or involved in initiation of the genome sequencing study
2.	Management of the research study:			
	Informed consent	No informed consent for secondary use in studies on schizophrenia, inbreeding, population migration Access to samples for other researchers without consent	No informed consent for secondary use in studies on genetic ancestry, drug abuse Access to samples for other researchers without consent	Integrity of consent in that the indigenous peoples did not understand the nature of the scientific study on account of illiteracy No collective consent from the San leadership
	Communication on and governance of research process	The tribal donors had no communication on or control of their samples as the samples were shared within the scientific community	The tribal donors had no communication on or control of their samples as the samples were shared within the scientific community	San leadership had no information on the genome sequencing and no input throughout the research process
	Transfer of samples/data	Samples transferred to researchers in	Samples were transferred to the Universities of Utah and Oxford without the consent of the tribal leaders	‐
3.	Publication of the research findings	On the secondary studies research findings that offended the cultural dignity of the Havasupai were published such as findings on inbreeding	On secondary studies in genetic ancestry and drug abuse that offended the cultural dignity of the tribe	Research findings published without approval of San leadership and no prior peer review of the research findings Use of language that was denigrating for the San peoples Publication of conclusions outside the genomic research results
4.	Termination of research studies:			
	Communication of research findings	Tribal leaders not informed that the study on diabetes did not reveal any genetic link and had been abandoned	Tribal leaders not informed that the study on arthritis did not show any genetic link and was terminated	No communication of the research findings to the San leadership
	Return of research samples	Returned in 2010 as part of an out of court settlement	Returned 20 years later as a result of protests by the tribe members	‐

## 3 INDIGENOUS PEOPLES’ HUMAN GENOMIC SOVEREIGNTY

### 3.1 Indigenous peoples’ sovereignty in international law and indigenous peoples’ data sovereignty

State sovereignty means a state's supreme authority within territory and signifies independence from any form of subservience to any higher authority. The key markers of state sovereignty are exclusive jurisdiction, territorial integrity, and centralized authority, while externally it is marked by non‐interference with its internal affairs. In the context of decolonisation, state sovereignty emblematically signified the prize of political independence for nation‐states in non‐Western societies, which was borne out of colonial conquest.^51^ The nation‐state was thus the internationally recognised model of political organisation and it denied power and nationhood status to tribal, cultural and other kinship arrangements with overlapping claims of territorial control.^52^ Essentially, state sovereignty reconstructed the competing notions of ‘nation’ to that based on the colonial conquest, while vanquishing any claim of nation status to any other forms of political organisation.

Indigenous peoples have been portrayed as part of the ‘unfinished business of decolonisation’ as the decolonisation process failed to legitimise the unsurrendered sovereignty of indigenous peoples within the newly established nation‐states.^53^ The notion of indigenous peoples’ sovereignty is thus an outcome of postwar‐decolonisation. As pointed out, the decolonisation process and emergence of sovereign nation‐states denied tribal, cultural, and other kinship arrangements nationhood status and placed them within the agency of the sovereign state. Though denied nationhood status, indigenous peoples’ pursued recognition as sovereign political collectivities within the nation‐state. The development of international human rights law stemming from the failures of the nation‐state in the treatment of its own citizens resulted in recognition of people as holders of rights independent of the nation‐state and regardless of the political organisation.^54^ Accordingly, the rights of ethnic, racial, religious, or other minority groups to pursue their political, economic, and socio‐cultural development within the state gained prominence. It is within the international human rights law paradigm of minority rights, right of peoples to self‐determination and right of people to enjoy their cultural heritage as a collective that the recognition of the sovereign prerogatives of distinct political, social, and cultural communities in international law resurfaced.^55^

The notion of indigenous peoples’ sovereignty in international law finds expression in the UN Declaration on the Rights of Indigenous People, 2007 (UNDRIP).^56^ The UNDRIP recognizes indigenous peoples right to internal collective self‐determination—autonomy to govern their internal and local affairs.^57^ This sovereignty is a derivative of the nation‐state sovereignty and is restricted within the domestic structures of the nation‐state.^58^ Therefore, the right to self‐determination of indigenous peoples’ is expressed within the sovereignty of the territorial state. The import of indigenous peoples’ sovereignty is that it bestows on indigenous peoples’ significant sovereign authority that would otherwise be exercised by the state. However, the sovereignty does not in any way equal the sovereignty exercised by the state—it is subordinate to— and in practice its’ exercise has been restricted to the territories occupied by indigenous communities and only in relation to their members.^59^ Indigenous peoples’ sovereignty thus only grants them certain rights that limit state sovereignty over their affairs.

Tied to the right to self‐determination is the indigenous peoples’ procedural right to participate through their own representatives in decision making in matters that would affect their rights, including their lands, territories, populations, and the resources therein. This right finds expression through the principle of free, prior and informed consent which is provided for in the UNDRIP.^60^ The principle concretises the indigenous peoples’ right to self‐determination as it secures their substantive participation and consent in matters concerning them.^61^ The UNDRIP expressly requires application the free, prior and informed consent in relation to: relocation of indigenous peoples from their lands; redress for cultural, religious, spiritual and intellectual property and for land, territory and resources taken from them unprocedurally; adoption and implementation of legislative and administrative measures affecting them; storage and disposal of hazardous material in their lands or territories; and projects affecting their lands and territories or other resources.^62^

In terms of substantive rights, the UNDRIP contexualises existing human rights principles to the historical circumstances of indigenous peoples. These include rights to self‐determination and self‐governance; culture, spirituality, and linguistic identity; participatory rights meant to redress their economic and social disadvantage; and land, territories, and resources.^63^ In the specific context of human genomic resources, articles 31 and 32 of the UNDRIP are instructive. Article 31 (1) recognises the rights of indigenous peoples to maintain, control, protect and develop their resources and their intellectual property over those resources.^64^ The UNDRIP specifically identifies human genetic resources, hence it forms the basis for indigenous peoples claim of collective proprietary rights to their human genetic material and data. Article 31 (2) spells out the corresponding state obligations which require states to recognise and protect control, maintain, and develop their resources.^65^ Article 32 gives indigenous peoples the right to prioritise development on and of their land, territories, and resources. In relation to this right the state has an obligation to consult and cooperate to obtain free, prior, and informed consent in prioritisation of projects affecting the lands, territories, or resources of indigenous peoples.^66^ Essentially, the UNDRIP refers to land, territories, and natural resources to reflect the totality of the relationships that indigenous peoples have with their lands and natural resources. In summary, the UNDRIP guarantees indigenous peoples the right to maintain cultural and spiritual relationships with their resources, the collective right to own, use, develop and control their natural resources and the right to free, prior, and informed consent in decisions concerning them or their resources.

Indigenous peoples’ data sovereignty stems from the right to self‐governance that accrues to indigenous peoples. The right to data sovereignty is anchored in Article 31 which provides for the indigenous peoples collective right to maintain, control, and protect their human and genetic resources, hence the right to govern data about their peoples’, lands or resources by choosing how, when, and how much control to exert. Generally, data sovereignty has been defined as management of information, which has been converted and stored in binary form, in line with the laws of the nation‐state in which it is located.^67^ Drawing from Article 31 of the UNDRIP, indigenous peoples data comprises: information and knowledge about their lands and territories and the cultural and religious relations between them and the territories; information about indigenous peoples; and information and knowledge about indigenous peoples as collectives.^68^ Indigenous peoples’ data sovereignty is thus defined as the right of indigenous peoples to govern collection, ownership and application of all their data, whether gathered by themselves or by external entities.^69^ In addition, under the principle of free, prior and informed consent, indigenous peoples should be consulted on who should gather data about them, what data should be gathered, who should maintain it and who should have access to it and what use it should be put to.

In human genomic research, indigenous peoples’ data sovereignty connotes the right to control ownership of and access to, use and reuse of human genetic data of indigenous peoples as individuals and as a collective whether generated by themselves, research institutions or government agencies.^70^ The claim of indigenous data sovereignty challenges the conceptions of western science and foregrounds issues of data collection, usage, and ownership. The open data culture that has been promoted within human genomic research since the HGP is at odds with indigenous peoples’ data sovereignty as based on their cultural and sacred beliefs, not all data is open to public access.^71^ This may in turn leave their data outside the open data infrastructure, hence denying them the benefits of genomic research. In addition, individuals are the generators of the data and have autonomy on whether or not to share it, while contrastingly, indigenous data sovereignty acknowledges data as part of indigenous group's collective identity, thus requiring collective consent for collection and usage and also invoking collective ownership by indigenous peoples.

Even then, the exercise of indigenous peoples’ data sovereignty is tied to the recognition and acknowledgment of indigenous peoples’ status as sovereign nations able to govern their lands, territories, people, and resources.^72^ As we demonstrate in the subsequent discussions, in practice the exercise of indigenous peoples’ sovereignty must compete with state sovereignty and often from a subordinate position.

### 3.2 Tribal and Aboriginal sovereignty in the USA and Canada

In this section we explore indigenous peoples’ sovereignty in the USA and Canada with a view to extrapolating its scope and practical application in human genomic research. The USA and Canada are similar in that both states cast negative votes against the UNDRIP in 2007,^73^ but in subsequent years have adopted policy positions and frameworks in support of the UNDRIP, promising to implement the indigenous peoples’ rights guaranteed in the UNDRIP.^74^

#### 3.2.1 Tribal sovereignty in the USA

Indigenous people in the USA are the Indian or Alaska native tribes, referred to as Indian tribes. The USA recognises the Indian tribes through the US Federal Indian policy.^75^ The US Constitution does not expressly mention Indian tribal sovereignty, hence Indian tribes are omitted in the dispersal of governmental authority between federal and state governments. The Constitution instead mentions Indian sovereignty in the commerce clause on regulation of commerce.^76^ The Indian tribes are mentioned alongside foreign nations and states, thus recognising the inherent authority of Indian tribes to self‐governance. The treaty clause of the Constitution also implicitly recognises Indian tribal sovereignty as it empowers the President to make treaties with them with the consent of the US Congress.^77^

The Indian tribes are thus considered domestic dependent nations, not by virtue of delegation of sovereignty, rather based on an acknowledgment of their existence as sovereign nations prior to European settlement.^78^ Though considered full sovereigns, who relate with the USA on a government to government basis, the sovereignty of the Indian tribes is subject to the federal authority.^79^ The tribal sovereignty extends to regulation of their own internal affairs in relation to their members and reservations/territories, but does not include external sovereign powers.^80^ As a marker of their inherent sovereignty, the tribes establish their own internal government, determine membership requirements, set up law enforcement and court systems in their own territories.^81^ To this end, the Indian tribes regulate their tribal economies, manage tribal cultural and natural resources, provide healthcare and educational needs for their members and exclude persons from their tribal lands.^82^ The federal government can exercise concurrent jurisdiction on tribal lands and can also diminish tribal jurisdiction through Congress.^83^ Indian tribes elect their leaders, who like other government leaders enjoy immunity in relation to official functions.^84^

In respect of the relationship between states and Indian tribes, states historically lack authority over the Indian tribal nations’ territories and members.^85^ The federal government is charged with protection of tribal nations from regulation and interference by states. On the other hand, the relationship between the tribal nations and the federal government is based on trust responsibility in which the US federal government undertakes to protect the Indian tribes and consult them before taking any action that impacts the tribal nations.^86^ The implication of the trust responsibility in part is that the Indian tribes by submitting to the protection of the USA relinquished some of their sovereignty. Accordingly, Congress has power to limit the sovereignty of Indian tribes through treaties and statutes.^87^ Fredericks III described the tribal sovereignty of the Indian tribes as unique and limited.^88^ It is unique to the extent that the Indian tribes have a government to government relationship with the USA and is limited in that by subjecting themselves to the protection of the USA, the Indian tribes ceded some of their sovereignty, such as the capacity to conclude treaties with foreign nations.^89^

In practice the nature of Indian tribes sovereignty is further been limited by the US Supreme Court which has since 1978, in its interpretation of Congress statutes, narrowed it to from territory‐based sovereignty to membership‐based.^90^ Moreover, the requirement of federal recognition limits the enjoyment of rights and protections for non‐recognised tribes as it is only through federal recognition of their existence that Indian tribes are able to establish government to government relationship, thus enabling them to enjoy rights and protections, particularly immunities and self‐governance.^91^ As of January 2024, 574 Indian tribes are federally recognised. The process of federal recognition is through: an administrative executive process; Congress; or the US courts. However, in practice recognition is mainly through the administrative executive process as the US courts have been reluctant to grant recognition, while the Congressional process is political and would involve lobbying.^92^ In addition, there are about 60 Indian tribes that are only state recognised, and do not enjoy federal recognition.^93^

In the context of human genomic research, tribal sovereignty finds expression in the ability of Indian tribes to regulate public health through passing of their own laws and regulations to operationalise health services for their members and within their territories and to promote health, for example, to commission and govern research studies on their members and in their territories. These expressions of sovereignty have extended to governance of research on indigenous people and governance of data through policies, laws and codes of conduct. For instance, the Havasupai tribe in exercise of tribal sovereignty issued a moratorium against biomedical research in their territory following the ASU case discussed above.^94^ Similarly, the Navajo nation has enacted the Navajo National Human Research Code which establishes the Navajo National Research Review Board mandated to review and approve research proposals. In addition, the Navajo National Human Research Code requires researchers to submit themselves to the Navajo civil jurisdiction in relation to the research to be undertaken and the research findings publications.^95^ Indian tribes also exercise sovereignty through control of their biological samples, for instance the Alaska Area Specimen Bank which is run by the Alaska Native Tribal Health Consortium.^96^ Nonetheless, Garba et al pointed out that for Indian tribes which are not federally recognised, the ability to exercise sovereignty over human genomics research is curtailed by lack of administrative capacity to put in place enforcement mechanisms and access to resources.^97^ This aptly demonstrates the limitation that the requirement for federal and state recognition of Indian tribes imposes on exercise of tribal sovereignty in the USA.

#### 3.2.2 Aboriginal sovereignty in Canada

In Canada indigenous peoples are referred to as aboriginal people and comprise of Indians, Inuit, and Métis peoples of Canada.^98^ The right to aboriginal people self‐government is protected in the Constitution.^99^ It guarantees the aboriginal people self‐governance in matters relating to their peoples and indigenous culture, but does not extend to a right to establish sovereign nations within Canada. The right stems from acknowledgment that the aboriginal people existed as self‐governing societies prior to European colonisation. Aboriginal sovereignty is governed by the fundamental principle of honour of the Crown, which personifies the special relationship between the aboriginal people and government of Canada which stemmed out of the assertion of Crown sovereignty over the aboriginal sovereignty that already existed prior to the Crown.^100^ The relationship embodied by the principle is unique and two sided: it seeks to reconcile the tension created by the unilateral imposition of laws by the Crown; and imposes an obligation on the Crown to act honourably in the dealings with the aboriginal people, treat them fairly, with respect and protect their rights.^101^ The obligation of the Crown to act honourably results in an imbalanced relationship in which the aboriginal people rely on the Crown for protection of their rights, with the overall effect of limiting aboriginal sovereignty.^102^ The Supreme Court of Canada has defined the instances in which the principle is invoked to include: discretionary control of aboriginal people interests; negotiation and interpretation of treaties; duty to consult and accommodate the interests of the aboriginal people when an act affects their rights and interests; and to diligently apply constitutional and statutory obligations relating to aboriginal people rights.^103^ The honour of the Crown principle beyond limiting aboriginal sovereignty has also been critiqued for lacking foundation in that it does not provide a persuasive explanation for extinguishing aboriginal rights and for ambiguity as the obligation of the Crown to act ‘honourably’ is imprecise.^104^

In relation to exercise of aboriginal sovereignty, Reinders identified the approaches to self‐governance to include: indigenous people governance on indigenous lands; creation of collective identity institutions; and indigenous and governmental partnerships.^105^ Indigenous people self‐governance on indigenous lands recognises territorial autonomy and is characterised by delegation of jurisdictional powers over indigenous territory. The delegated powers include regulating activities over the territory, business licensing, planning and zoning, administration of local justice and environmental protection.^106^ However, indigenous self‐governance based on territorial autonomy is limited as only those living in the territory can exercise and enjoy self‐governance, while those indigenous people living outside indigenous lands do not. The second approach of self‐governance entailing institutional arrangements shifts from territory‐based autonomy to autonomy based on indigenous people relationships and is characterised by establishment of institutions that govern indigenous people relationships with each other and their lands.^107^ For instance, the author highlights the Toronto Native Care and Child Services which provides family care services based on the cultural values of indigenous people. The third approach entails local, provincial, or federal level partnerships with indigenous communities, which provide avenues for shared governance. The shared governance is based on agreements that take into account indigenous sovereignty. The shared governance agreements extend to service provision, land and resource management, tax collection, health, and policing services.^108^

In relation to human genomic research, aboriginal people sovereignty has been exercised through enactment of laws and regulations to regulate research on their territories and land. For instance, the Nuu‐chaah‐nulth people established a research ethics committee to control research on and about their members.^109^ In addition, the aboriginal people have shared jurisdiction with the provincial governments to enforce the laws relating to health matters. However, unlike the Indian nations in the USA, a number of initiatives meant to concretise indigenous peoples’ human genomic sovereignty have been undertaken by the government with the participation of aboriginal people. First is the 2000 establishment of the Institute of Aboriginal Peoples’ Health which supports and regulates health research on and involving aboriginal people.^110^ Second, is the development of the Canadian Guidelines for Health Involving Aboriginal People which were an offshoot of the Nuu‐chaah‐nulth case and were developed with the participation of aboriginal people.^111^ These guidelines recognise the culture and jurisdiction of the aboriginal people in collection and application of their human genetic resources and data.^112^ In addition, the guidelines ensure exercise of sovereignty in ownership of the human genetic resources through the concept of DNA on loan, in which researchers return genetic material and turn in data if the aboriginal people require it.^113^

### 3.3 Indigenous peoples’ sovereignty in Africa

The debate on indigenous peoples’ sovereignty in Africa had first to contend with the ‘indigenousness question’: is it relevant in Africa? The roots of this debate can be traced to: first, the political project of nation‐state building of post‐colonial African states which aimed to establish national identities based on the dominant national cultures.^114^ The effect was political reluctance to recognise groups whose cultures differed from the dominant cultures resulting in marginalisation and subjugation of the culturally distinctive groups.^115^ Second, and tied to the political project was that the discourse on the rights of indigenous peoples at the global level evolved based on Western contexts that conceptualised indigenousness in ways that did not resonate with African realities. The Western concept of indigenousness emphasised precedence over territory before colonisation in its definition.^116^ The effect was that African post‐colonial governments countered the ‘indigenousness question’ by pointing out that all Africans were natives of the continent prior to colonisation.^117^

Over the last two decades, the African regional human rights system has affirmatively answered the ‘indigenousness question’ in Africa by contexualising the concept to resonate with realities of culturally distinctive groups in the Continent. Accordingly, the African Commission on Human and Peoples’ Rights (African Commission) reconceptualised indigenousness in Africa by shifting from the emphasis on territorial precedence prior to colonisation to: cultural distinctiveness from the dominant state cultures; attachment to ancestral lands and natural resources for survival; discrimination based on social status— adherence to their cultural and religious practices and beliefs; and marginalisation and exploitation in the political and economic systems.^118^ Applying this criteria in the *Endorois* case, the African Commission further clarified that the key markers of indigenousness for the Endorois were self‐identification and attachment to ancestral lands and natural resources therein.^119^

At the African Union political level, the position on the indigenousness question in Africa can be observed from the political positions taken by the African political leadership. A good starting point is the position taken by African states in the drafting of the UNDRIP. First, in 2006 the African Union (AU) opposed the draft UNDRIP through a non‐action resolution sponsored by Namibia, thus deferring consideration of the draft UNDRIP by the General Assembly.^120^ Second, in January 2007 the AU assumed a united front against the draft UNDRIP and called for further consultations in relation to: definition of indigenous peoples; scope of self‐determination; ownership of land and resources; national and territorial integrity; and establishment of distinct economic and financial institutions.^121^ Third, the opposition by African states resulted in compromises in the draft UNDRIP which qualified self‐determination to the right to autonomy of indigenous peoples in their own local and internal affairs and the introduction of a provision expressly prohibiting of external self‐determination. The effect of the express prohibition of external self‐determination was that it subordinated indigenous peoples’ sovereignty to the Westphalian (national) sovereignty.^122^

African states were concerned about the implication of an unqualified right to self‐determination of indigenous peoples on national sovereignty and territorial integrity. For instance, Rwanda in its submission to the drafting committee of the draft UNDRIP indicated that the UNDRIP isolated certain groups and prompted them to establish alternative institutions outside the central authority of the sovereign state.^123^ These concerns are contextually rooted in the history of colonisation in Africa and its continued legacy post‐colonisation. On colonisation and its legacy post‐colonisation, Wiessner noted that while decolonisation purported to afford colonised people in Africa political independence through the establishment of sovereign nation‐states, this independence was given to people within geographical demarcations determined by the European colonisers, rather than to individual societies as constituted prior to colonisation.^124^ The import is that to date African sovereign states are politico‐geographical entities whose citizens were constituted by European colonisers.^125^At decolonisation, the UN deployed the accepted international law doctrine of *uti possidetis* meaning that colonial boundaries were to be retained as demarcated at the time of colonisation.^126^ Ultimately then, political independence did not accrue to colonised populations but to people within borders determined by European colonisers. This position remains to date. The implications are twofold: first for populations in Africa the geographical demarcation of political boundaries did not take into account distinct linguistic and cultural ethnic groups so that single and identical ethnic groups were divided by the colonial boundaries and are today dispersed across different nation‐states. Second, for states, these dispersal of identical groups across nation‐states was a threat to the nation‐state national identity building project, giving birth to politics of inclusion and exclusion within the Continent.

Post‐colonisation the AU accepted the doctrine of *uti possidetis* as a pillar of African political governance,^127^ meaning that colonial boundaries in Africa are sacrosanct regardless of their arbitrariness in splitting apart identical ethnic groups and bundling together hostile and non‐identical ethnic groups. In international law, the doctrine of *uti possidetis* does not imply that colonial boundaries are inviolable, rather the boundaries may be renounced by common agreement. In the case of Africa, Mnyongani noted that African leaders twice had occasion to reconsider Africa's colonial boundaries.^128^ First, he noted that the 1958 All Africa Peoples Conference, a non‐governmental assembly of political and trade union leaders, rejected the colonial boundaries and called for their readjustment. Five years later, during the formation of the Organisation of African Unity (OAU), post‐independence African leaders elected to maintain the colonial boundaries to safeguard the stability of the Continent.^129^ Second, in 2002, during the transition of the OAU to the AU, African leaders were once again confronted with the issue and elected to retain the colonial boundaries, a position which was enshrined as one of the principles of the African Union.^130^ Given the multi‐ethnic nature of Africa, readjustment of colonial boundaries along ethnic groupings would undoubtedly destabilise the Continent and pose challenges to political unity transcending ethnic differences.

### 3.4 Comparing the exercise of indigenous peoples’ sovereignty in the USA, Canada, and in Africa

The above discussion of indigenous peoples’ sovereignty in Africa points to both commonalities and differences with the earlier discussed sovereignty of tribal nations in the USA and the aboriginal people in Canada. The marked commonality is the practice of the Westphalian state to limit the exercise of indigenous peoples’ sovereignty through introduction of administrative bureaucracy requirements and restrictive interpretation of the scope of indigenous sovereignty by domestic courts in the USA and Canada, and in Africa political reluctance in many states to give effect to indigenous peoples’ rights. In the USA, as noted above the US Supreme Court has over the years limited the exercise of the sovereignty of tribal nations through interpretive decisions that have chipped at territorial sovereignty and restricted its application to their members. In addition, the requirement of federal recognition of Indian tribes limits protection and exercise of tribal nations’ rights by the federal government. In Canada, the honour of the Crown principle subordinates aboriginal sovereignty to the Crown sovereignty based on the unequal political and economic power and has also been interpreted by the domestic courts restrictively, for instance the restrictive definition of the Crown, which absolves municipal agencies from the obligations attaching to the principle.^131^ Further, in practice the self‐governance of the aboriginal people is restricted to only those members living on indigenous lands. In Africa, the continued political reluctance to give effect to the exercise of indigenous peoples’ sovereignty in many states can for instance be observed in the failure to implement decisions of the African human rights institutions on the rights of indigenous peoples. It is possible to argue that these decisions are both against one state— Kenya, and also that non‐implementation of decisions of the African Court and the Commission abounds in relation to other rights. However, viewed broadly from the perspective of the continued rampant violation of indigenous peoples’ rights, such as forced displacement of from their lands, cultural suppression and political and economic marginalisation,^132^ a picture of political non‐recognition of their rights in the continent emerges.

On the differences in the exercise of indigenous sovereignty, two inter‐linked differences stand out: (i) the sanctity that attaches to national sovereignty in Africa; and (ii) effect of political‐geographical boundaries on self‐governance of indigenous groups across nation‐state borders in Africa. On the sanctity that attaches to national sovereignty in Africa, as alluded to earlier, during decolonisation, independence for African states was viewed as the ultimate prize of the colonial conquest. Drawing from this, African states place a high premium on national sovereignty and territorial integrity. As discussed earlier, African leaders in the 1963 OAU Charter unequivocally protected the sovereignty, territorial integrity and right to independence of the newly independent African states.^133^ In this regard, any challenge to national sovereignty is frowned upon and African states cannot countenance overlapping and parallel sovereignties. This informed the joint position taken by the African Union in January 2017 against the draft UNDRIP—the African political leadership could not accept parallel sovereignties. In addition, while in the U.S. and Canada the Indian tribes and the aboriginal people signed treaties with the sovereign state at independence and continue to negotiate agreements with their governments, in the African context at independence the ‘nation’ was constituted based on colonial borders drawn by the colonisers with the effect that inhabitants within certain geographical borders were constituted into the ‘nation’. This means that African states only construe sovereignty as national sovereignty emanating out of their independence— there are no known sovereign polities in any particular territory prior to independence. This explains why the notion of indigenous peoples’ sovereignty at the national level remains deeply contested and without much political acknowledgment and recognition in Africa. It thus puts assertions of indigenous sovereignty in Africa at a much weaker position compared to the USA and Canada.

On the political‐geographical boundaries and their effect on self‐governance of the indigenous peoples in Africa, while indigenous people retain their genetic make‐up regardless of these geographical boundaries, self‐governance is at the core of their exercise of human genomic sovereignty. To underscore the limitations posed by the political‐geographical boundaries, Hodgson, writing on the negotiation of the UNDRIP, noted in relation to the Maasai of East Africa that the colonial boundaries between the Maasai of Kenya and those of Tanzania posed practical coordination problems in terms of presenting a collective front relating to their recognition as an indigenous group.^134^ In Africa, indigenous people are dispersed across nation‐states borders, for instance, as earlier discussed, the San people of Southern Africa are scattered in Namibia, Botswana, Lesotho, Angola, South Africa, Swaziland, and Zimbabwe; the East African Maasai are scattered in Kenya and Tanzania; and the Amazigh of North Africa across Morocco and Algeria. The effect of these political boundaries is that it weakens collective coordination among indigenous people and the ability to establish self‐governance mechanisms in exercise of their sovereignty. Without the ability to coordinate and establish self‐governance mechanisms, the protracted question of who is the representative of indigenous authority of these groups across various nation‐states arises. Contrastingly, the Indian nations in the USA and the aboriginal people in Canada have specific territory which they occupy and exercise self‐governance over those territories and their members. Indigenous peoples in Africa lack exclusive and defined territory due to being split apart and this poses complex problems to indigenous peoples in exercise of their right to autonomy and local/internal self‐governance, since even if they share cultural and linguistic similarities, their dispersal across nation‐states results in weakened coordination. As observed in the San people case, these political boundaries mount collective coordination problems since even though the San Council claimed to represent all the San people of Southern Africa, the San Council does not enjoy political recognition and its geographical reach extends only to the San people of South Africa. Notably, the Namibia government approved research on the San people of Namibia without any reference to the group leadership.

## 4 LESSONS FOR AFRICA ON INDIGENOUS PEOPLES’ HUMAN GENOMIC SOVEREIGNTY

In this final section, we revisit the overarching question of the article, on the indigenous peoples’ human genomic sovereignty in Africa. Borrowing from indigenous peoples’ data sovereignty, indigenous peoples’ human genomic sovereignty entails a right of indigenous peoples to govern and control the collection, ownership and use of data derived from their biological specimen and human sequences. The exercise of genomic sovereignty as discussed in the Indian tribes and aboriginal people practice entails governing of human genomic research in their territories and among their members through: promulgation of laws and policies; approval of research studies, including granting collective permission; exercise of civil and criminal jurisdiction over researchers; controlling access to and use of human genetic material and data, including return of samples; and ownership over human genetic resources. In Africa and as illustrated in the San people case study the contextual realities point to the need for political recognition of indigenous peoples’ sovereignty, and collective coordination mechanisms among indigenous peoples groups.

At the outset, Caroll et al noted that acknowledgment and recognition of indigenous peoples’ sovereignty by the nation‐state (Westphalian state) is at the core of the exercise of indigenous peoples’ data sovereignty.^135^ As earlier discussed, in Africa recognition and acknowledgment of indigenous peoples’ sovereignty at the political level is lacking, which at the national level presents as denial of indigenous peoples’ status as political entities with sovereign prerogatives and rights. This lack of recognition and acknowledgement of their sovereignty effectively denies them the power to enact and enforce laws governing their human genomic data and to exercise jurisdictional control over human genomic research involving their members. Garba et al in their analysis on indigenous peoples’ research governance in the USA emphasised the importance of political recognition by pointing out that Indian nations without federal recognition lack the administrative capacity to exercise self‐governance over human genomic research involving them. Therefore, political recognition is key for the exercise of human genomic sovereignty in Africa. As noted earlier, the African Commission has had remarkable success in contexualising the concept of indigenousness in Africa for the legal recognition of indigenous peoples’ rights. To address the lack of political recognition, the African Commission should clarify the concept of indigenousness in Africa further, and elaborate the concept in relation to new emerging rights such as indigenous peoples’ data sovereignty. The African Commission has developed a collection of African centric guidelines that elaborate on implementation of certain rights, and accordingly, it should develop guidelines to further clarify on indigenousness in Africa and also to elaborate on the rights of indigenous peoples in the specific context of human genomic data. In essence, these guidelines will serve two key purposes: provide indigenous peoples with soft law norms for reasserting human genomic data and research sovereignty at national level; and as a restatement of state obligations in relation to indigenous peoples’ data sovereignty.

In relation to the political‐geographical boundaries that pose collective coordination problems and weaken the ability to establish self‐governance mechanisms, the USA and Canada experiences, discussed above, allude to the fact that the indigenous people are able to govern research on their members, even outside their territorial lands. However, this must be viewed in context— that indigenous peoples in the USA and Canada enjoy government recognition, hence exercise sovereignty through enacting laws that govern research, including issuing moratoriums, demanding return of samples and data collected unlawfully and imposition of fines and penalties. The San people case in which the Government of Namibia authorised research on the San people in Namibia without any regard to the San Council illustrates the need for recognition and acknowledgement of the representative authority of the indigenous peoples self‐governance structures by national authorities and among the indigenous peoples groups. Hudgson noted in the context of UNDRIP negotiation that colonial borders introduced notions of political inclusion and exclusion among similar cultural and linguistic groups which contributed to the inability of indigenous peoples in Africa to form regional and continental networks.^136^ Given the legal recognition of various indigenous groups in Africa across national borders, these groups should establish coalitions and networks that transcend national borders and have proper self‐governance structures that clearly designate their representatives. With particular reference to human genomic research, the coalitions and networks should develop codes of research conduct and set out the obligations to researchers. Notably, following the San people case, the San Council adopted the San Code of Research Ethics. In addition, there is need to educate their members across national borders of the existence of the research codes.

Further, on the dispersal of indigenous people in Africa across national borders, the indigenous peoples regional networks should leverage on the regional economic communities in Africa to push for their political recognition at the sub‐regional level. Even though split across national borders, often similar indigenous groups fall within the same regional economic community, for instance the Maasai of Kenya and Tanzania and the Batwa of Uganda and Democratic Republic of Congo are in the East African Community; the San people spread throughout six Southern Africa states are in Southern Africa Development Community; while the Amazigh of Morocco and Algeria are in the Arab Maghreb Union. Indigenous peoples groups should politically mobilise and leverage on the political institutions of the regional economic communities for recognition and to advance their interests. In practice the regional economic communities have developed human rights frameworks to protect the rights of their citizens on matters that are common to the sub‐region and require a coordinated approach. For example, the Economic Community of West African States adopted a declaration against human trafficking in 2012 to provide a framework for the sub‐regional states to address human‐trafficking from West Africa.^137^ Borrowing from this, indigenous peoples groups can collectively mobilise beyond national orders for political recognition at the sub‐regional level and for adoption of human rights instruments that elaborate on their rights in relation to human genomic research.

Finally, protecting and promoting the right to participate in and to enjoy the benefits of scientific progress and its applications (the human right to science) in Africa to facilitate indigenous peoples’ agency in self‐governance in human genomic research. While the African Charter on Human and Peoples’ Rights does not expressly provide for the right, it provides for the right of everyone to take part in the cultural life of their community. The human right to science has been described as a cultural right encompassing the right to participate in scientific life, hence it finds implicit protection in the African Charter.^138^ The human right to science includes participation in the progress of science.^139^ For indigenous peoples in Africa to have their genomic data represented in genomic databases, they require the necessary agency to enable them understand the complexities of human genomic research and thus exercise human genomic sovereignty to collectively protect their cultural heritage. As observed in the San people case, the four elders representing the San people in Namibia did not understand what they consented to and how it collectively related to the rights of San people as a collective. As a starting point on the human right to science, the African Commission should adopt a resolution explaining the rights, its contours in human genomic research and other emerging data science applications, the state obligations and its application in relation to indigenous peoples in Africa.

## CONFLICT OF INTEREST STATEMENT

The author declares no conflict of interest.

## Footnotes

1 Resnik, D.B. (1999). Human Genome Diversity Project: Ethical Problems and Solutions. Politics and the Life Sciences. 18 (1): 15‐23.

2 Ibid.

3 Harry, D. (2008). Indigenous Peoples and Gene Disputes. Chicago‐Kent Law Review. 84 (1), 147‐196.

4 International Human Genome Sequencing Consortium. (2001). Initial Sequencing and Analysis of the Human Genome. Nature. 409: 860‐921.

5 Ibid.

6 Harry, op. cit. note 3, pp. 189‐191.

7 Ibid: 183‐184.

8 Hodgson, D.L. (2009). Becoming Indigenous in Africa. African Studies Review. 52 (3): 14.

9 Garrison, N.A., Hudson, M., Ballantyne, L.L., Garba, I., Martinez, A., Taualii, M., Arbour. L., Caron, N.R., & Rainie, S.C. (2019). Genomic Research through an Indigenous Lens: Understanding the Expectations. Annual Review of Genomics and Human Genetics. 20:495‐517; Van Assche, K., Gutwirth, S., & Sterckx, S. (2013). Protecting Dignitary Interests of Biobank Research Participants: Lessons from *Havasupai Tribe v Arizona Board of Regents*. Law Innovation and Technology. 5(1):55‐85.

10 Van Assche, et al., op. cit. note 9, p.58.

11 Ibid.

12 Ibid.

13 Reardon, J., & TallBear, K. (2012). “Your DNA is Our History”: Genomics, Anthropology and the Construction of Whiteness as Property. Current Anthropology. 53(5):233‐245.

14 Ibid.

15 Garrison, et al., op. cit. note 9, pp. 498‐499.

16 Ibid.; Van Assche, et al., op. cit. note 9, p.62.

17 Van Assche, et al., op. cit. note 9, p.61.

18 Van Assche, et al., op.cit. note 9, p.62.

19 Ibid.; Reardon & TallBear, op. cit. note 13, p.241.

20 Wiwchar, D. (2004, December 16). Nuu‐chaah‐nulth Blood Returns to West Coast. Ha‐Shilth‐Sa, 31(25). p. 1.

21 Dalton, R. (2002). Tribe Blasts ‘Exploitation’ of Blood Samples. Nature. 420: 111.

22 Schimdt, C.W. (2001). Indi‐Gene‐Ous Conflicts. Environmental Health Perspectives. 109(5):216‐219.

23 Ibid.

24 McInnes, R.R. (2011). Culture: The Silent Language Geneticists Must Learn— Genetic Research with Indigenous Populations. The American Journal of Human Genetics. 88: 254‐261.

25 Dalton, op. cit. note 21.

26 Wiwchar, op. cit. note 20, p. 1.

27 Dalton, op. cit. note 21.

28 Wiwchar, op. cit. note 20, pp. 3‐4.

29 Ibid.

30 Ibid.; McInnes, op. cit. note 24, p. 256.

31 McInnes, op. cit. note 24, p. 258.

32 Botes, M.W. (2017). Visual Communication as Legal‐Ethical Tool for Informed Consent in Genome Research Involving the San Community of South Africa. Unpublished PhD Thesis, University of South Africa. (p. 11‐12). (on file with author).

33 Chennells, R., & Steenkamp, A. (2018) International Genomics Research Involving the San People. In D. Schroeder, J. Cook, F. Hirch, S. Fenet, & V. Muthuswami (eds.), Ethics Dumping: Case Studies from North‐South Collaborations (pp. 15‐22). Cham, Switzerland: Springer Nature Publishing.

34 Brӧrmann, M. (2010, April 28). The Kalahari San, WIMSA. Cultural Survival. Retrieved January 8, 2024, from https://www.culturalsurvival.org/publications/cultural-survival-quarterly/wimsa; Chennells, R., & Schroeder, D. (2019). The San Code of Research Ethics. In D. Schroeder, K. Chatfield, M. Singh, R. Chennells, & P. Herissone‐Kelly (Eds.), Equitable Research Partnerships: A Global Code of Conduct to Counter Ethics Dumping (pp. 73‐87). Cham, Switzerland: Springer Nature Publishing.

35 South African San Council. (2023). Promoting and protecting the rights of the San. Retrieved January 8, 2024, from https://www.sancouncilsa.org/.

36 Chennells & Steenkamp, op. cit. note 33, pp. 17‐20.

37 Schuster, S.C., Miller, W., Ratan, A., Tomsho, L.P., Giardine, B., Kasson, L.R., et al. (2010). Complete Khoisan and Bantu Genomes from Southern Africa. Nature. 463(7283): 943‐947.

38 Ibid.

39 Ibid.

40 Ibid.

41 Botes, op. cit. note 32.

42 Ibid.

43 Chennells & Steenkamp, op. cit. note 33, p.17.

44 Ibid.

45 Schlebusch, C. (2010). Issues Raised by Use of Ethnic‐Group Names in Genome Study. Nature. 464(7288):487.

46 Chennells & Steenkamp, op. cit. note 32, pp. 18‐19.

47 Botes, op. cit. note 31, pp. 234‐235.

48 Chennells & Steenkamp, op. cit. note 32, p. 18.

49 Chennells & Steenkamp, op. cit. note 32, pp. 20‐21; Chennells & Schroeder, op. cit. note 33, pp. 73‐87.

50 Ibid.

51 Oguamanam, C. (2004). Protecting Indigenous Knowledge in International Law: Solidarity beyond the Nation‐State. Law Text Culture. 8 (10): 189‐230.

52 Ibid.

53 Ibid.

54 Ibid.

55 Wiessner, S. (2008). Indigenous Sovereignty: A Reassessment in Light of the UN Declaration on the Rights of Indigenous People. Vanderbilt Law Review. 41(4): 1141‐1176.

56 United Nations General Assembly. (2007). UN Declaration on the Rights of Indigenous Peoples. A/RES/61/295. Retrieved January 12, 2024, from https://www.un.org/development/desa/indigenouspeoples/wp-content/uploads/sites/19/2018/11/UNDRIP_E_web.pdf.

57 Ibid: Article 4.

58 Ibid.

59 Wiessner, op. cit. note 55, p. 1167.

60 United Nations General Assembly, op. cit. note 55, Article 19.

61 Hofbauer, J.A. (2022). Free, Prior and Informed Consent (FPIC). In C. Binder, M. Nowak, J.A. Hofbauer, & P. Janig (Eds.), Elgar Encyclopedia of Human Rights (pp. 249‐259). Cheltenham, UK: Edward Eglar Publishing.

62 United Nations General Assembly, op. cit. note 55.

63 Ibid.

64 Ibid.

65 Ibid.

66 Ibid.

67 Ougamanam, C. (2020). Indigenous Peoples, Data Sovereignty, and Self‐determination: Current Realities and Imperatives. The African Journal of Information and Communication. 26: 1‐20.

68 Russo Caroll, S., Garba, I., Figueroa‐Rodríguez, O.L., Holbrook, J., Lovett, R., Materechera, S., et al. (2020). The CARE Principles for Indigenous Data Governance. Data Science Journal. 19 (43): 1‐12.

69 Russo Caroll, S., Rodriguez‐Lonebear, D., & Martinez, A. (2019). Indigenous Data Governance: Strategies from the United States First Nations. Data Science Journal. 18 (31): 1‐15; The First Nations Information Governance Centre. (2019). The First Nations Data Sovereignty in Canada. Statistical Journal of the IAOS. 35: 47‐65.

70 Russo Caroll, et al., op. cit. note 68, p. 3.

71 Ougamanam, op. cit. note 67, p. 5.

72 Russo Caroll, et al., op. cit. note 69, p. 3.

73 United Nations. (September 13, 2007). General Assembly Adopts Declaration on the Rights of Indigenous Peoples; ‘Major Step Forward’ towards Rights for All, Says President. Retrieved January 12, 2024, from https://press.un.org/en/2007/ga10612.doc.htm.

74 US Department of State, Office of the Tribal Justice. (January 12, 2011). Announcement of US Support for the United Nations Declaration on the Rights of Indigenous Peoples. Retrieved January 12, 2024, from https://2009-2017.state.gov/documents/organization/154782.pdf; Government of Canada, Department of Justice. (2018). Principles Respecting the Government of Canada's Relationship with Indigenous Peoples. Retrieved January 12, 2024, from https://www.justice.gc.ca/eng/csj-sjc/principles.pdf.

75 Cohen, F.S. (2012). Cohen's Handbook of Federal Indian Law. New York: LexisNexis.

76 United States Senate. (September 13, 1787). Constitution of the United States of America, Article 1, §8, cl. 3. Retrieved January 12, 2024, from https://www.senate.gov/about/origins-foundations/senate-and-constitution/constitution.htm.

77 Ibid: Article 2, §2, cl. 2.

78 Cohen, op. cit. note 75.

79 Ibid.

80 Ibid.

81 Ibid.

82 Ibid.

83 Ibid.

84 Ibid.

85 Ibid.

86 Ibid.

87 Ibid.

88 Fredricks III, J. (1999). America's First Nations: The Origins, History and Future of America Indian Sovereignty. Journal of Law and Policy. 7 (2): 347‐410.

89 Ibid.

90 Doran, M. (2020). Redefining Tribal Sovereignty for the Era of Fundamental Rights. Indiana Law Journal. 95 (1): 87‐144.

91 Congressional Research Service. (2024, January 18). The 574 Federally Recognised Tribes in the United States. Retrieved June 24, 2024, from https://crsreports.congress.gov/product/pdf/R/R47414#:~:text=AfederallyrecognizedTribeis,forarecognizedTribeCincluding.

92 Ibid.

93 US Department of Health and Human Sciences. (2024). Administration for Native Americans Fact Sheet. Retrieved June 2024, from https://www.acf.hhs.gov/ana/comms-fact-sheet/ administration‐native‐americans

94 Harry, op. cit. note 3.

95 Ibid.

96 Reardon & TallBear, op. cit. note 13.

97 Garba, I. Stering, R., Plevel, R., Carson, W., Cordova‐Marks, F., Cummins, J., et al. (2023). Indigenous Peoples and Research: Self‐determination in Research Governance. Frontiers in Research Metrics and Analytics. 8 (1272318), 2.

98 Government of Canada, Justice Laws. (January 1, 2021). The Constitution Act, 1982, §35, cl. 2. Retrieved January 12, 2024, from https://laws-lois.justice.gc.ca/eng/const/page-13.html#docCont.

99 Ibid: §35, cl. 1&3.

100 Centre for Constitutional Studies, Honour of the Crown. (August 4, 2021). Retrieved June 24, 2024, from https://www.constitutionalstudies.ca/2021/08/honour-of-the-crown/.

101 Ibid.

102 Ibid.

103 Ibid.

104 Ibid; Slattery, B. (2005). Aboriginal Rights and Honour of the Crown. Supreme Court Law Review. 29, 433‐445.

105 Reinders, K. (2019). A Rights based Approach to Indigenous Sovereignty, Self‐determination and Self‐government in Canada. SURG: Studies by Undergraduate Researchers at Guelph. 19: 1‐11.

106 Ibid.

107 Ibid.

108 Ibid.

109 Wiwchar, op.cit. note 20, pp.3‐4.

110 McInnes, op. cit. note 24, pp. 258‐259.

111 Ibid.

112 Canadian Institutes of Health. (2013). CIHR Guidelines for Health Research Involving Aboriginal People. Retrieved January 12, 2024, from https://cihr-irsc.gc.ca/e/29134.html.

113 Ibid.

114 Hodgson, op. cit. note 8, pp.1‐3.

115 Ibid.

116 Ibid: pp.7‐8; Gilbert, J. (2011). Indigenous Peoples’ Human Rights in Africa: The Pragmatic Revolution of the African Commission on Human and Peoples’ Rights. The International and Comparative Law Quarterly. 60 (1): 249‐251.

117 Ibid.

118 African Commission on Human and Peoples’ Rights. (2006). Report of the African Commission Working Group of Experts on Indigenous Communities/Populations. Retrieved June 25, 2024, from https://achpr.au.int/en/special-mechanisms-reports/report-african-commissions-working-group-experts-indigenous.

119 African Commission on Human and Peoples’ Rights. (2009). Centre for Minority Rights Development (Kenya) and Minority Rights Group (on Behalf of Endorois Welfare Council) v Kenya, Communication 276/03 (Merits Judgement) para 162. Retrieved January 13, 2024, from https://achpr.au.int/en/decisions-communications/centre-minority-rights-development-kenya-and-minority-rights-group-27603.

120 Gover, K. (2015). Settler‐State Political Theory, ‘CANZUS’ and the UN Declaration on the Rights of Indigenous Peoples. European Journal of International Law. 26(2):345‐373; Davis, M. (2012). To Bind or Not to Bind: The United Nations Declaration on the Rights of Indigenous Persons Five Years On. Australian International Law Journal. 19 (3):17‐48.

121 Engle, K. (2011). On Fragile Architecture: UN Declaration on the Rights of Indigenous Peoples in the Context of Human Rights. European Journal of International Law. 22 (1):141‐163.

122 Bauder, H., & Mueller, R. (2023). Westphalian Vs. Indigenous Sovereignty: Challenging Colonial Territorial Governance. Geopolitics. 28(1):156‐173.

123 Davis, op. cit. note 120, pp. 22‐23.

124 Wiessner, op. cit. note 55, p. 1150.

125 Ibid.

126 Ibid: 1151.

127 African Union. (2001). Constitutive Act of the African Union, art. 4(b). Retrieved January 13, 2024 from https://au.int/sites/default/files/pages/34873-file-constitutiveact_en.pdf.

128 Mnyongani, F.D. (2008). Between a Rock and a Hard Place: The Right to Self‐determination versus *Uti Possidetis* in Africa. Comparative and International Law Journal of Southern Africa. 41(3): 463‐479.

129 Ibid:466‐467.

130 Ibid:467‐468.

131 McMorrow, T. (2018). Upholding the Honour of the Crown. Windsor Yearbook of Access to Justice. 35, 328‐333.

132 Violations Against Indigenous Africa. University of Michigan. Cases. Retrieved June 26, 2024, from https://indigenousafrica.org/browse-cases/countries/. *See cases* of cultural suppression of the Amazigh in Algeria and Morocco; forced displacement of indigenous peoples in Uganda and Tanzania for the East African crude oil pipeline project; displacement of the Batwa from the Katanga region in Democratic Republic of Congo; economic and cultural suppression of the Afar in Djibouti and the Bassari in Senegal.

133 African Union. (1963). Organization of African Union Charter, Article 3(3). Retrieved January 13, 2024, from https://au.int/sites/default/files/treaties/7759-file-oau_charter_1963.pdf.

134 Hodgson, op. cit. note 8, pp.15‐16.

135 Caroll, op. cit. note 69, p. 3.

136 Hudgson, op. cit. note 8, pp. 13‐14.

137 African Commission on Human and Peoples’ Rights. (2012). ECOWAS Declaration on the Fight Against Trafficking in Persons. Retrieved June 26, 2024, from https://achpr.au.int/en/node/856.

138 Bidault, M. (2022). Considering the Right to Enjoy the Benefits of Scientific Progress and its Applications as Cultural Right: A Change in Perspective. In H. Porsdam, & S.P. Mann (Eds.), The Right to Science: Then and Now (p. 142). Cambridge, UK: Cambridge University Press.

139 United Nations Committee on Economic, Social and Cultural Rights. (2020). General Comment No. 25 (2020) on Science and Economic, Social and Cultural Rights (Article 15 (1) (b), (2), (3) & (4) of the International Covenant on Economic, Social and Cultural Rights. Retrieved June 26, 2024, from https://www.ohchr.org/en/documents/general-comments-and-recommendations/general-comment-no-25-2020-article-15-science-and.

